# Depression after Stroke in Sub-Saharan Africa: A Systematic Review and Meta-Analysis

**DOI:** 10.1155/2017/4160259

**Published:** 2017-07-27

**Authors:** Akin Ojagbemi, Onoja Akpa, Fisayo Elugbadebo, Mayowa Owolabi, Bruce Ovbiagele

**Affiliations:** ^1^College of Medicine, University of Ibadan, Ibadan, Nigeria; ^2^University College Hospital, Ibadan, Nigeria; ^3^Kwame Nkrumah University of Science and Technology, Kumasi, Ghana

## Abstract

**Objective:**

We aimed to conduct a systematic review and meta-analysis of prevalence and characteristics of poststroke depression (PSD) in sub-Saharan Africa (SSA).

**Methods:**

We searched Medline, PsycINFO, and African Journals OnLine using keywords for stroke and depression and the .mp. operator for all 54 SSA countries/regions. Further information was retrieved through a manual search of references from relevant published and unpublished articles. We included only peer-reviewed original studies with epidemiological or experimental designs, conducted random-effect meta-analysis, and identified the most commonly associated factors by weight (inverse of variance method).

**Results:**

Seventeen studies, comprising 1483 stroke survivors, met the criteria for syntheses. The pooled frequency of clinically diagnosed PSD was 31% (95% CI = 26%–36%), versus 13.9% in healthy control pairs. Prevalence did not vary much across healthcare settings but was affected by methods of depression ascertainment. PSD was significantly associated with low education, cognitive impairment, physical disability, poor quality of life, and divorced marital status.

**Conclusion:**

Almost 1 in 3 individuals with stroke in SSA has clinical depression. Despite limitations around quality of identified studies, results of the present systematic review overlap with findings in the global literature and highlight useful targets for the design and trial of tailored intervention for PSD in SSA.

## 1. Introduction

There is an escalating burden of stroke in sub-Saharan Africa (SSA), which is now among the leading causes of disease and disability in the region [1]. A key complication of stroke is depression, which while being associated with tremendous poststroke morbidity is frequently under-recognized and undertreated, especially in SSA, where resources are relatively limited and the issues of mental health are underappreciated.

A recently published scientific statement on poststroke depression (PSD) by the American Heart Association/American Stroke Association (AHA/ASA) [2] highlighted the current state of evidence regarding scientific knowledge and clinical practice of PSD. However, there was only one study from SSA [3] out of a combined total of 236 citations in the five systematic reviews [4–8] in the AHA/ASA statement.

The limited data on PSD in SSA in the AHA/ASA scientific statement was likely due to the publication of African studies in typically less visible and less accessible media, such as databases of African literature. Yet, extrapolating results derived from a global pool of studies to SSA may mask the true state of burden of depression in stroke survivors living in the subregion.

The objective of this study was to conduct a systematic review and meta-analysis of PSD studies among patients in SSA to arrive at a pooled estimate of prevalence and to identify the qualitative and quantitative relationships of key clinical characteristics with occurrence of PSD.

## 2. Methods

This review followed conventional recommendations for the methodology and reporting of systematic reviews [9, 10].

### 2.1. Search Strategy

The search strategy aimed to find both published and unpublished studies. An initial search of the African Journal OnLine (12 February 2017; repeated 14 May 2017), Medline (Ovid SP 1946—15 February 2017; repeated 14 May 2017), and PsycINFO (Ovid SP 1806—16 February 2017) databases was conducted using the following keywords with the “explode” operator: stroke OR poststroke OR “cerebrovascular accident” OR “Cerebrovascular disorder” OR “stroke survivors” AND depression OR “major depression” OR “major depressive disorder” OR “depressive symptoms” OR “poststroke emotional disorders” OR “emotional response” AND epidemiology OR frequency OR prevalence OR incidence OR factors OR “risk factors” OR “associated factors” OR “precipitating factors” OR “predisposing factors” OR outcome OR mortality. We next used the .mp. operator to search each of the 54 SSA countries or regions by name.

A second stage consisting of hand searching of the reference list of relevant articles retrieved from the databases was also implemented.

Limits on language and publication dates were not imposed in conducting the searches.

### 2.2. Inclusion Criteria

#### 2.2.1. Types of Participants

We included studies involving stroke survivors regardless of the method of stroke diagnoses in both the qualitative and quantitative components of the review.

#### 2.2.2. Types of Phenomena of Interest

We included studies investigating epidemiological phenomena such as frequencies, prevalence, incidence, risk or associated factors, and outcome in both the qualitative and quantitative components of the review.

#### 2.2.3. Types of Studies Included

We have the following:

- Studies where participants were recruited from their homes (herein and after referred to as community studies), hospital, rehabilitation settings, nursing homes, or other such institutions.
- Studies with epidemiological or experimental study designs. This included descriptive and analytical cross-sectional studies, prospective and retrospective cohort studies, case control studies, randomized controlled trials, nonrandomized controlled trials, quasi-experimental studies, and before and after studies.

### 2.3. Exclusion Criteria

We have the following:

- Nonhuman literature
- Review papers
- Case series and individual case reports
- Studies focusing solely on qualitative data
- Other textual materials such as expert opinion, discussion papers, and position papers.

### 2.4. Study Assessments and Data Extraction

Study assessment for inclusion and exclusion criteria as well as subsequent data extraction was conducted by two independent assessors based on the descriptions in the original article. It was agreed a priori that in cases of disagreement, a consensus will be reached based on the decision of an experienced colleague.

#### 2.4.1. Ascertainment of Risk of Bias in Studies Exploring Associations

A standard framework for assessing biases in studies investigating associations between variables [9, 11] was used for judgments about the risk of bias in studies describing associations. All 5 steps in the modified Graphical Appraisal Tool for Epidemiologic (GATE) studies [9, 11] were used for the determination of the risk of bias. We determined external validity by assessing key characteristics of the eligible sample in the relevant studies and made judgments about the level of representativeness of the source population. We made judgment about internal validity by assessing the method of identification of stroke survivors, outcome measurements, and analytical strategies. These steps were undertaken to ensure that the associations identified by the respective studies are valid and are not due to unidentified factors that may be related to both exposure and outcome.

Risk of bias was classified as low, unclear/unknown, and high [9]. Points were allocated to each component of the study as follows: 2 points when the risk of bias was low, 1 point when this was unclear/unknown, and no points when the risk of bias was clearly high. Judgment about overall risk of bias in the selected studies was made by averaging risk of bias for a particular study and calculated by summing up the total points accrued by that study and dividing the result by the total number of components assessed. Finally, we classified the overall risk of bias for a particular study as high (when the average risk of bias scores for that study is less than 1), moderate (when this is between 1 and 1.5), and low (when the score is greater than 1.5).

### 2.5. Statistical Methods

Meta-analysis was conducted using prevalence estimates of PSD reported in the original articles meeting the criteria for quantitative synthesis. We centered the display of prevalence estimate on a point of zero for the purpose of illustration.

As heterogeneity was expected due to differences in the type of depression assessments (diagnostic or rating scales), as well as setting of studies, a random-effect meta-analysis model was chosen. To reduce the extent of methodological heterogeneity, we combined studies with similar diagnostic procedures in the same meta-analysis model. To determine the extent of statistical heterogeneity, we estimated the percentage of total variation in estimates reported across studies that is due to heterogeneity, rather than chance. This was computed using the *I*^2^ test. Values of *I*^2^ > 50% are often regarded as evidence of statistical heterogeneity [12]. In considering clinical heterogeneity, additional subgroup meta-analyses based on the setting of the studies (i.e., medical inpatients, outpatients, or physical rehabilitation) were conducted.

For the objective of investigating the most important factors associated with PSD by rank, we used the log of odds ratios (OR) and the corresponding standard errors (SE) of the associations. The inverse of variance method was used for weighing in all quantitative estimations.

All quantitative analyses were conducted using the “*metan*” add-on in Stata version 12.0 [13] and Cochrane review manager (Revman) version 5.3 software [14].

## 3. Results

### 3.1. Search Results

The combined database, unpublished literature, and hand searches identified a total of 74 records. After removing duplicates in either database (*N* = 8  articles), the titles and abstract of 18 articles were screened. The full texts of all 18 articles were retrieved. After reading through the texts, one article from South Africa [15] was excluded as depression was not directly measured. The authors used a visual analogue scale to measure broadly defined emotional reactions in stroke survivors with aphasia.

**Figure 1:**
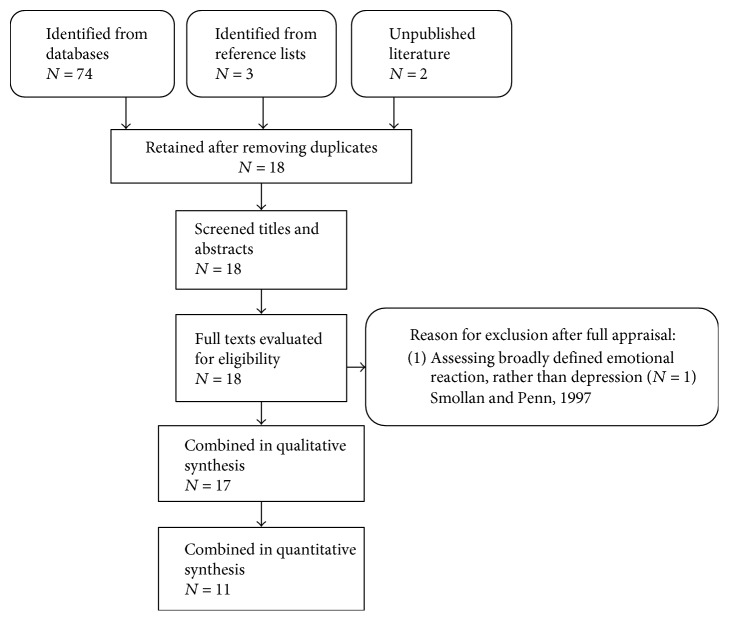
Flow chart showing details of included and excluded studies.

Studies included were conducted between October 1998 and January 2017. Other details of included and excluded studies are shown on the flow chart in Figure 1.

### 3.2. Qualitative Appraisal of Identified Studies

#### 3.2.1. Geographical Location of Studies

All included studies came from primarily two regions in sub-Saharan Africa: West Africa and East Africa. However, about 65% of identified studies were from one country, Nigeria.

#### 3.2.2. Types of Study Design and Methods

Ten of the 17 included studies were of cross-sectional designs, two studies included stroke survivors and healthy control pairs [16, 17], and the remaining 5 were prospective observational studies of between 2- and 12-month duration [18–22].

While many studies were set up to explore prevalence (*N* = 11) (Table 1) and associated factors (*N* = 9), only very few investigated relationship of depression with stroke outcomes (*N* = 5) [17, 18, 21, 23, 24]. None of the studies investigated incidence of PSD or the risk factors for new onset of the disorder.

Four [19, 25–27] of the 9 studies investigating cross-sectional associations with PSD cited factors without systematically investigating their association with the disorder (i.e., they did not use appropriate analytical techniques in establishing associations). The remaining five studies described the source population and sampling frame. They also provided estimates of OR or sufficient data to allow for such estimation. However, two [28, 29] of these five studies had limitations of sample size. The combined risk of bias, as assessed using the modified GATE criteria, in the five studies reporting factors that are systematically associated with poststroke depression was moderate.

#### 3.2.3. Assessment for Depression

All included studies used valid methods for the ascertainment of depression. While most relied on a variety of rating scales, clinical interview and formal diagnostic criteria were used in five studies [16, 24, 26, 28, 30]. Only one of these studies [30] used the criteria in the 10th revision of the International Classification of Diseases (ICD-10) [31]. The other studies using clinical diagnostic criteria relied on the Diagnostic and Statistical Manual of Mental Disorders [32].

#### 3.2.4. Healthcare Settings of Studies

Nearly one-half (47.1%) of the studies were conducted in physical rehabilitation settings. Notably, only one study [17] included participants recruited from their homes, rather than in hospital or physical rehabilitation settings. The remaining eight studies were conducted among medical inpatients [20–22] or outpatients [3, 25, 27, 30].

#### 3.2.5. Participant Characteristics

Most of the identified studies did not specify types or severity of stroke, nor were detailed exclusion criteria reported. Two studies included participants who were within a 6-month poststroke period [20, 22]. The other studies were heterogeneous in terms of poststroke periods investigated.

### 3.3. Quantitative Syntheses

#### 3.3.1. Prevalence of PSD

**Figure 2:**
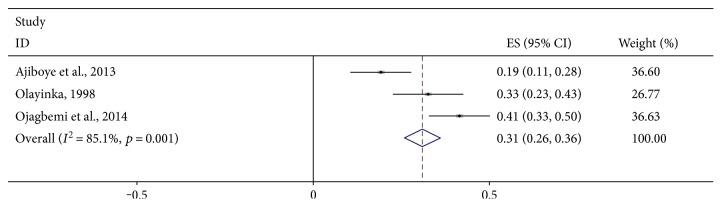
Forest plot showing the pooled prevalence of clinically diagnosed poststroke depression in sub-Saharan Africa.

**Figure 3:**
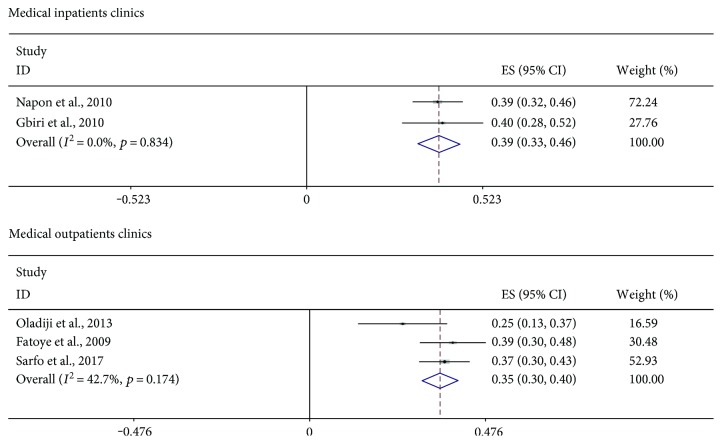
Forest plots showing the pooled prevalence of poststroke depression (using rating scales) in medical inpatient/outpatient clinics.

**Figure 4:**
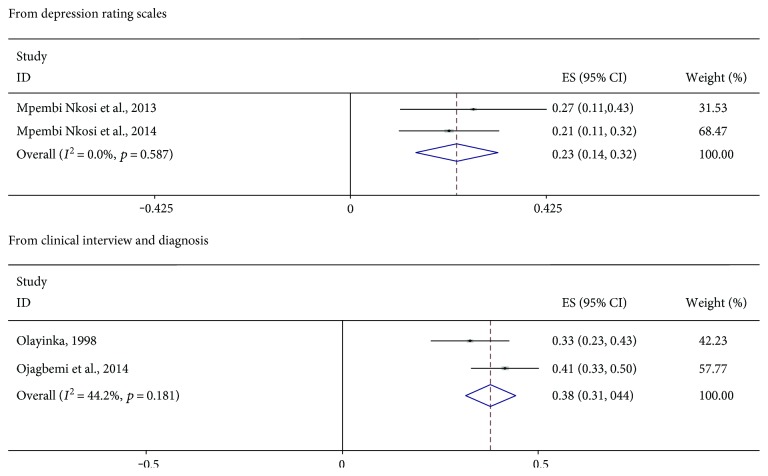
Forest plots showing the pooled prevalence of poststroke depression in the rehabilitation setting.

Figures 2 and 3 are forest plots showing the prevalence of depression in studies meeting the criteria for quantitative syntheses. The 95% confidence intervals of each estimate together with their quantitative summary are also presented. Greater weights are given to studies with narrower confidence intervals. The pooled frequency of clinically diagnosed PSD was 31% (95% CI = 26%–36%). There was an indication of statistical heterogeneity in this estimate (*I*^2^ = 85.1%, *p* < 0.001). This was due to one study [30] reporting an unusually low prevalence of PSD in a Nigerian medical outpatient clinic (Figure 2). That study was the only study in the present review using the ICD-10 criteria. It is unclear whether, or how, this ascertainment procedure might have affected the results of that study [30]. In analyses conducted to minimize heterogeneity (Figures 3 and 4), there were no remarkable differences in pooled rates reported across healthcare settings. The highest pooled frequency (39%) was found among medical inpatients. However, the prevalence of PSD in the studies appears to be dependent on ascertainment procedure. For example, using rating scales in rehabilitation settings, a pooled frequency of 23% was estimated, whereas formal diagnostic assessments in the same healthcare setting produced a pooled frequency of 38% (Figure 4). The frequency of depression in healthy control pairs was found to be 13.9% in the only study [16] including such participants.

#### 3.3.2. Cross-Sectional Associations with PSD

The factors with the most precise systematic association with PSD, by weight and in decreasing order, were low levels of education, cognitive impairment, physical disability, and divorced marital status (Table 2). However, the most frequently cited factors in studies were female gender and physical disability. Though highly cited, the association of female gender with PSD was weakened by inflated SEs in studies reporting this association.

#### 3.3.3. Association of Depression and Stroke Outcomes

Poor quality of life, which was the most frequently cited outcome of stroke linked to depression, was systematically investigated in only one multivariate analysis (Table 3). Although poor sexual health and functional dependency after stroke were also linked to depression, only functional dependency was investigated in a multivariate analysis.

## 4. Discussion

In conducting a comprehensive pooled review of the prevalence and characteristics of PSD in SSA, key observations were made. The pooled frequency of clinically diagnosed PSD in SSA is 31%. Prevalence did not vary much across healthcare settings but appeared to be affected by methods of depression ascertainment (diagnostic assessments or rating scales). PSD was significantly linked to cognitive and functional deficits, as well as low levels of education and divorced marital status. Depression in this population was also linked to poor quality of life and functional dependency after stroke.

The observations in the present review are made within limitations of the quality of available data. There were many small studies. These studies were also very different in the types of healthcare settings of stroke survivors. For example, stroke survivors in medical inpatient settings, physiotherapy, and community can be expected to have varying degrees of morbidity. Additional differences in the study were the use of a variety of methodologies in the ascertainment of both stroke and depression in the identified studies, thus limiting a meaningful inclusion of many studies in our quantitative syntheses without compromising heterogeneity. Given these limitations, well-designed studies, especially including prospective observation, adequate sample sizes, well-defined cohorts of stroke survivors, are still required to better situate the evidence-practice gap of PSD in SSA.

Despite the above limitations, the pooled frequency of PSD reported in the present review overlaps with findings from the two largest systematic reviews estimating the global prevalence of PSD. In the review by Hackett et al. [5] including 51 studies (reported in 96 publications) globally before June 2004, the pooled frequency of PSD was 33% (95% CI = 29%–36%). In the subsequent systematic review by Ayerbe et al. [6] including 43 cohorts published before August 2011, a pooled frequency of 29% (95% CI = 25%–32%) was reported. We note that these large systematic reviews of the global literature on the PSD did not include about 95% of the studies identified in the present investigation.

We were unable to provide robust data depicting the natural history of PSD by estimating incidence and prevalence across different poststroke periods because most of the studies identified in the present systematic review were cross-sectional in design.

Even though the global literature suggests that both biological and psychological factors are pathophysiologically linked to PSD [2], our data suggests that, in SSA, the disorder has so far been associated with reduced social and economic potential to cope with (physical and cognitive) disability. We note that as in many low- and middle-income countries, levels of formal education and marital status are important proxies of social and economic positions in SSA [33]. In this regard, having little formal education and no spousal support may be associated with social and economic disadvantages.

Biological factors that have been associated with PSD in the global literature include problems with the mechanisms of cerebral blood flow [34], neurotransmitters, and inflammatory cytokines [35], as well as gene polymorphism [36]. In the only study identified in the present review where the association between depression and stroke types, lesion, and their brain locations was investigated [28], these biological factors showed no cross-sectional association with depression (lesion characteristic OR = 0.4, 95% CI = 0.0–6.2). Similar to the global evidence [2], PSD in SSA has also been systematically linked with poor quality of life and functional dependency after stroke.

In reviewing the existing evidence, a number of gaps in the SSA literature on PSD have emerged that are of potential relevance for the design of such future studies.

### 4.1. The Incidence and Natural History of PSD in SSA

Globally, about one-third of stroke survivors will experience depression at some point in the poststroke period [4]. The evidence suggests that rates of new onset depression do not vary much in the first year poststroke. The five observational studies identified in the current systematic review missed an opportunity to estimate the incidence of PSD in their sample. Given the unique social, economic, and cultural circumstances in SSA, it is yet unclear whether rates of new onset depression after stroke may vary one way or another compared with reported rates from other parts of the world.

### 4.2. Biological Factors Contributing to the Pathophysiology of PSD in SSA

Studies from other parts of the world suggest that PSD is not merely a psychological response to disability [35, 36] as several biological factors have been implicated in the pathophysiology of the disorder. As earlier stated, we identified one small study investigating whether stroke lesion characteristics are associated with PSD [28]. That study cross-sectionally analyzed the computerized tomography images of 30 patients and found no relationship between stroke lesions and depression. As such, the relevant biological factors for PSD in survivors living in SSA remain to be investigated.

### 4.3. Effective Interventions for PSD in SSA

The global literature suggests that a wide range of effective pharmacological and nonpharmacological interventions have been designed and tested for the prevention and treatment of PSD in other parts of the world [2]. Studies aiming to design and test tailored interventions for the prevention or treatment of PSD are yet to be conducted in SSA. The finding in the present systematic review, suggesting that measures of reduced social and economic potential to cope with (physical and cognitive) disability may be important in the pathophysiology of PSD, provides important leads for the design and testing of culturally relevant and tailored intervention for the disorder in the subregion.

#### 4.4. Strength and Limitations

The search strategy was designed to be meaningfully sensitive. In this regard, the searches have focused on some of the largest repository of biomedical literature with additional strategy to cover African and unpublished literature, including manual searches of the references of the appraised articles. Nevertheless, the interpretation of our results is limited by the small number of good-quality studies of association of several factors with PSD.

## 5. Conclusion

The pooled frequency of clinically diagnosed PSD in SSA is 31%. It is linked to functional dependency and poor quality of life after stroke. These findings overlap with reports in the global literature. However, PSD in SSA has so far been cross-sectionally associated with indices of reduced social and economic advantages in the face of cognitive and functional deficits. While there are subsisting evidence gaps precluding meaningful estimate of the full extent of the burden, the findings in the present systematic review provide useful targets for the design and trial of tailored intervention for the prevention and treatment of PSD in the subregion.

## Figures and Tables

**Table 1:** Prevalence of poststroke depression in sub-Saharan Africa.

Reference	Country/location	Type of study	Definition of depression	Sample size	Female (%)	Age mean (SD)	Time since stroke	Prevalence (%)
*Medical inpatient clinics*								
Napon et al., 2010	Burkina Faso	Observational (2 months)	Montgomery and Asberg Depression Rating Scale (MADRS).	167	52.3	56.9 (29–84)^a^	2 weeks	38.9
Gbiri et al., 2010	Nigeria	Observational (6 months)	Center for Epidemiological Study of Depression Scale (CES-D)	65	49.2	58.1 (15.7)	0–6 months	40.4
*Medical outpatient clinics*								
Oladiji et al., 2009	Nigeria	Cross-sectional	Depression, Anxiety and Stress Scale	51	39.2	52.5 (5.9)	5–18 months	25.2
Fatoye et al., 2009	Nigeria	Cross-sectional	Beck's Depression Inventory	118	42.4	59.6 (10.5)	1–24 months	38.8
Ajiboye et al., 2013	Nigeria	Cross-sectional	Schedules for Clinical Assessment in Neuropsychiatry (SCAN)/ICD-10	83	55.4	60.6 (13.2)	3–84 months	19.2
Gyagenda et al., 2015	Uganda	Cross-sectional	Patient Health Questionnaire-9	73	58.9	57.3 (20–99)^a^	NR	31.5
Sarfo et al., 2017	Ghana	Cross-sectional	Center for Epidemiological Study of Depression Scale (CES-D)/Geriatric Depression Scale (GDS)	200	47.5	62 (52–72)^a^	12–48 months	36.5
*Physical rehabilitation settings*								
Olayinka, 1998	Nigeria	Cross-sectional	Psychiatric Assessment Schedule (DSM III-R)	86	40.7	55.4 (9.1)	0–72 months	32.6
Mpembi Nkosi et al., 2013	Democratic Republic of Congo	Observational (1 year)	Patient Health Questionnaire (PHQ9)	30	30.0	55.9 (12.7)	NR	26.7
Mpembi Nkosi et al., 2014	Democratic Republic of Congo	Cross-sectional	Patient Health Questionnaire (PHQ9)	56	37.5	54.5 (12.6)	60.7% > 1 year	21.4
Ojagbemi et al., 2014	Nigeria	Case-control	Schedules for Clinical Assessment in Neuropsychiatry (SCAN)/DSM-IV	130	53.9	59.5 (11.1)	3–24 months	41.5

NR = not reported; ^a^range.

**Table 2:** The most common factors associated with poststroke depression in sub-Saharan Africa.

Independently associated factors	Estimated standard errors of association	Weight (%)
Low education^a^	0.04	38.8
Cognitive impairment^b^	0.08	37.6
Physical disability^c^	0.34	21.0
Divorced marital status^a^	1.41	2.4
Apathy^b^	13.3	<0.1
Female gender^c^	20.7	<0.1
Poor self-reported health status^a^	76.7	<0.1

Cited, not systematically associated	Reference	Sample size
Personal history of mental illness	Olayinka, 1998	86
Family history of mental illness	Olayinka, 1998	86
Poor social support	Olayinka, 1998	86
Right-side laterality	Oladiji et al., 2009	51
Older age	Mpembi Nkosi et al., 2014	30
Sleep disturbance	Mpembi Nkosi et al., 2014	30

^a^One study; ^b^two studies; ^c^four studies.

**Table 3:** Outcome of poststroke depression in sub-Saharan Africa.

Reference	Country/location	Type of study	Definition of depression	Outcome	Effect estimate	Effect (yes/no)
Howitt et al., 2011	Tanzania	Cross-sectional	Hospital Anxiety Depression Scale	Poor quality of life	−0.4^a^	Yes
Gbiri and Akinpelu, 2012	Nigeria	Observational (12 months)	Center for Epidemiological Study of Depression Scale (CES-D)	Poor quality of life	−0.8^b^	Yes
Akinpelu et al., 2013	Nigeria	Cross-sectional	Beck's Depression Inventory	Poor sexual satisfaction; poor ejaculation	454.5^c^	Yes
					236.5^c^	Yes
Ojagbemi and Owolabi, 2013	Nigeria	Cross- sectional	Schedules for Clinical Assessment in Neuropsychiatry (SCAN)/DSM IV	Functional dependency	3.1^b^	Yes
Hamza et al., 2014	Nigeria	Observational (12 months)	Beck's Depression Inventory	Poor quality of life	−0.2^d^	Yes

^a^Pearson correlation coefficient; ^b^adjusted odds ratio in logistic regression; ^c^Mann–Whitney *U* test; ^d^beta for multiple linear regression.
